# Rotator Cuff Repair Augmentation by Direct Interlocking of an Overlayed Nonwoven Polyethylene Terephthalate Patch Substantially Increases Repair Strength: A Biomechanical Comparison in Ovine Shoulders

**DOI:** 10.1177/23259671251356629

**Published:** 2025-07-21

**Authors:** Bettina Hochreiter, Ronja Senn, Elias Bachmann, Jess G. Snedeker, Karl Wieser

**Affiliations:** † Department of Orthopaedics, Balgrist University Hospital, University of Zurich, Zurich, Switzerland; ‡ Institute for Biomechanics, ETH Zurich, Zurich, Switzerland; § ZuriMED Technologies AG, Zurich, Switzerland; Investigation performed at the Balgrist Campus, Balgrist University Hospital, Zurich, Switzerland

**Keywords:** rotator cuff repair, patch augmentation, biomechanics, ex vivo ovine model

## Abstract

**Background:**

Despite advancements in surgical treatment of rotator cuff tears, such as the implementation of patches to reinforce repairs, the rate of retears remains high. Construct failure often occurs at the suture-tendon interface.

**Purpose/Hypothesis:**

The purpose of this study was to compare the biomechanical properties of 3 types of rotator cuff repair (RCR): a nonaugmented transosseous-equivalent (TOE) repair, a conventional patch–augmented TOE repair, and a TOE repair augmented with a polyethylene terephthalate (PET) patch that directly interlocks with the underlying tendon across its entire interface. It was hypothesized that interlocked patch augmentation of RCR is biomechanically superior to conventional TOE.

**Study Design:**

Controlled laboratory study.

**Methods:**

A total of 18 ovine infraspinatus tendons were detached, repaired (with TOE), and tested in 3 groups (n = 6): (1) nonaugmented TOE, (2) conventional patch–augmented TOE, and (3) interlocked patch–augmented TOE. In the second group, a commercial synthetic polyester patch was attached to the tendon via No. 2 FiberWire sutures and laterally attached to the humerus using No. 2 FiberWire sutures and 2 knotless anchors. In the third group, an interwoven patch-tendon interface was created using a microblade to push the fibers of the patch directly into the underlying tissue and to the humerus as described above. Each specimen underwent cyclic loading, followed by pull-to-failure testing. Ultimate tensile strength, cyclic and linear stiffness, peak-to-peak elongation, and gap formation were measured.

**Results:**

Direct patch interlocking resulted in significantly higher tendon purchase during pull to failure (587 ± 109 N vs 222 ± 48 N and 211 ± 52 N) as well as cyclic stiffness testing (44 ± 3 N/mm vs 25 ± 2 N/mm and 29 ± 2 N/mm) compared with the conventional patch–augmented and nonaugmented TOE, respectively (*P* < .0001 for all comparisons). Linear stiffness was also significantly higher compared with the conventional patch–augmented TOE (34 ± 6 N/mm vs 22 ± 2 N/mm; *P* = .007).

**Conclusion:**

While limiting but not eliminating tendon retraction, augmentation of a conventional TOE with direct interlocking of a nonwoven PET patch provided biomechanically superior results compared with conventionally augmented and nonaugmented TOE RCRs. The interlocked patch not only significantly improved time-zero force to failure but, compared with a conventional commercial patch design, also increased linear stiffness.

**Clinical Relevance:**

Higher construct stiffness suggests that micromotion and gap formation were minimized, an aspect that is crucial for tendon-bone healing and for reducing early tendon retraction, thereby offering potential to improve retear rates in future clinical applications.

Despite advances in the surgical management of rotator cuff tears (RCTs), retear rates remain high. They are commonly reported between 25% and 35% and as high as 94% for massive tears.^7,13,16^ Failure is most often due to sutures’ cutting through the tendon tissue, making the suture-tendon interface the most vulnerable part of the rotator cuff repair (RCR).^13^

One approach to address this issue and improve surgical outcomes is the use of a patch acting as a biological and/or mechanical reinforcement of the repair construct to enhance its healing capacity.^1,4,9^ This approach aims to achieve a superior RCR in terms of biomechanics. It includes strength and stiffness closer to that of a native tendon, resistance to gap formation, and optimization of the contact area and pressure, thereby facilitating successful tendon-bone healing.^19^ Numerous authors have studied patch augmentation for RCR with mixed results. Many have found improved biomechanical and clinical outcomes in patch-augmented groups compared with nonaugmented controls or preoperative conditions.^5,21,22,24,33,36,38,39^ However, there remains a lack of consensus regarding optimal patch placement and fixation technique. Grafts are commonly secured to the structurally weak myotendinous junction with conventional sutures and to the bone with conventional bone anchors or by stapling the patch to the tissue.^9,17,18,21,25,37^

Recently, a surgical approach was described for attaching a nonwoven polyethylene terephthalate (PET) patch to soft tissue.^29^ This technique involves a reciprocating microblade that carries individual patch fibers into the tendon tissue, creating an interwoven fiber network. The strong bond formed by the tissue-integrated patch fibers strengthens the interface by distributing the transfer of forces between the patch and the tendon over a larger area.

The purpose of this study was to evaluate and compare the biomechanical properties of a transosseous equivalent (TOE) RCR augmented through directly interlocking a nonwoven PET patch to a conventional patch–augmented TOE and a nonaugmented TOE. We hypothesized that this interlocked patch–augmented RCR would be biomechanically superior to conventional repair techniques.

## Methods

A total of 18 fresh sheep shoulders (n = 6/group), aged 6 to 8 months, were dissected fresh to remove all tissue except the humeri and the infraspinatus tendons (ISPs). These tendons are commonly used as RCR models because of their biomechanical and microscopic similarity to human supraspinatus tendons.^‖^ The ISPs were cut approximately 10 cm from their bony insertion point, visually inspected for abnormalities, and all muscle tissue was carefully removed. The humeri and ISPs were then stored in a cloth soaked in phosphate-buffered saline (PBS) at −20°C until use. Each specimen was allowed to thaw at 7°C for 12 hours prior to instrumentation and testing. Once thawed, the specimens were kept hydrated with PBS throughout testing.

### Surgical Technique

All RCRs were performed by a single, fellowship-trained shoulder surgeon (B.H.). The ISPs were sharply detached from the greater tuberosity with a scalpel to mimic a complete full-thickness tear and expose the approximately 1 × 2–cm footprint. Each specimen was randomly assigned to one of the repair groups.

### Control Group (1): Nonaugmented TOE RCR

**Figure 1.**
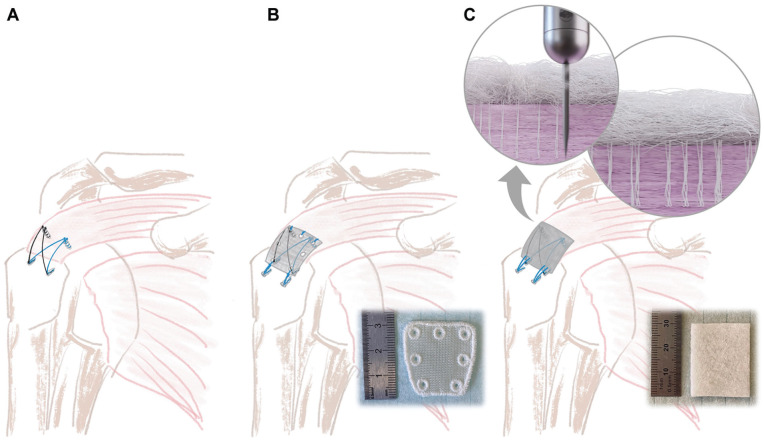
(A) Nonaugmented TOE, (B) conventional patch–augmented TOE, and (C) directly interlocking, nonwoven PET patch–augmented TOE. Circles in the top right corner illustrate the process of interlocking the patch with the underlying tendon. PET, polyethylene terephthalate; TOE, transosseous equivalent.

**Figure 2.**
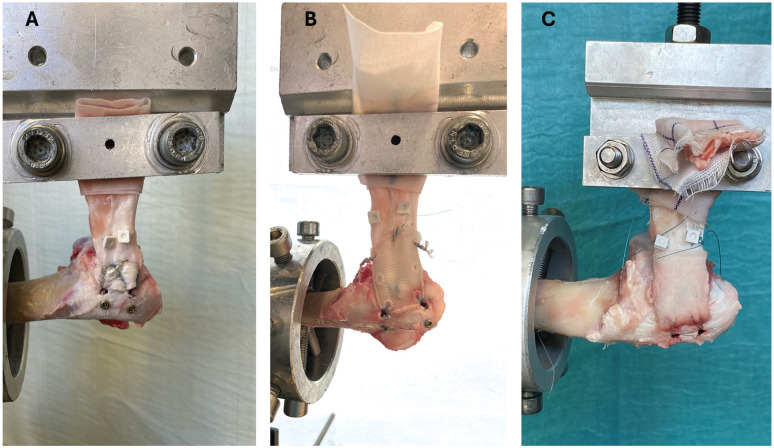
Biomechanical setup of the repair constructs using a materials testing machine. (A) Nonaugmented TOE RCR, (B) conventional patch–augmented TOE RCR, and (C) interlocked patch–augmented TOE RCR. RCR, rotator cuff repair; TOE, transosseous equivalent.

For each of the 18 specimens, a TOE RCR, currently considered the gold standard for RCRs from a biomechanical point of view,^10^ was performed using the same technique (Figures 1 and 2). For the medial row, two 5.5-mm fully threaded Corkscrew FT II anchors (Arthrex) were used, double-loaded with No. 2 FiberWire sutures (Arthrex). The medial bone anchors were inserted into predrilled holes along the medial border of the native footprint. All 8 suture limbs were passed through the tendon in a horizontal mattress configuration. The medial row was tied with a sliding double half hitch knot, followed by alternating simple half hitches for a total of 5 throws. The suture limbs of the medial row were used to bridge and compress the repaired tendon. Lateral fixation was performed with 2 knotless 4.5-mm ReelX STT (Stryker).

### Control Group (2): Conventional Patch–Augmented RCR

Following the initial TOE repair as described above, in 6 specimens, a commercially available synthetic polyester patch (Pitch-Patch; Xiros), measuring 3 × 2 cm with 7 reinforced fixation holes was placed on top of the TOE, covering the tendon as well as the lateral row anchors (Figures 1 and 2). Fixation was performed according to the manufacturer’s guidelines. Medially, 1 No. 2 FiberWire was passed through each of the 3 reinforced holes and the rotator cuff and secured with a surgeon’s knot followed by 5 alternating half hitches. The midposterior and midanterior fixation holes were not used since they exceeded the width of the ISPs. The lateral fixation was performed with 2 knotless 4.5-mm ReelX STT anchors, which were placed lateral to the lateral row, and No. 2 FiberWire sutures.

### Experimental Group: Directly Interlocking, Nonwoven PET Patch–Augmented RCR

In the remaining 6 specimens, the RCR augmentation was performed using a SpeedPatch PET patch (ZuriMED Technologies AG), a textile felt made of PET filament yarn, measuring 2.5 × 3 cm. As with the conventional patch–augmented control group, the patch was placed on top of the TOE, covering the tendon as well as the lateral row anchors (Figures 1 and 2). Two No. 2 FiberWire sutures were first passed laterally through the patch in a mattress stitch configuration and fixed to the humerus again using the same 2 knotless anchors, lateral to the lateral row. Prior to lateral fixation, patch-tendon attachment was achieved using a technique based on physical interlocking of nonwoven patch fibers into the underlying tissue by way of a reciprocating microblade (felting). The microblade features design elements (very small barbs) that catch and carry groups of loose fibers within the patch into the tendon. The FiberLocker Instrument (ZuriMED Technologies AG) runs at a frequency of 42 Hz and was applied for 90 seconds, moving the instrument over the entire patch surface to achieve a roughly uniform application to the tendon surface. The patch was attached to the tendon across the entire patch-tendon interface, which enhanced load sharing between the tendon and the patch (compared with conventional patches, usually sutured to specific locations on the tendon^9,17,18,25,26^).

After completing surgical steps, markers were attached to the tendon and bone for subsequent measurements of gap formation. Two custom-made 3-dimensionally printed markers were sutured to the tendon with 6-0 Ethilon sutures (Ethicon) approximately 5 cm medial to the tendon end (Figure 2). Two small screws were inserted just lateral to the most lateral row of bone anchors, to serve as reference markers on the humeral bone.

### Biomechanical Testing Protocol

The RCR constructs were tested using a universal testing machine (Zwick 1456; ZwickRoell) with a 20-kN load cell, with data recorded using its dedicated software (testXpert III; ZwickRoell). The humeri were placed in a custom cylindrical clamp, with the humeral shaft oriented horizontally to replicate the physiological pull direction of the native ISP in sheep, maintaining a 90° angle between the humeral shaft and the force vector (Figure 2). The ISPs were wrapped in a piece of cloth and clamped in a custom-designed soft tissue clamp that prevented slippage, leaving approximately 5 cm between the repair site and the soft tissue clamp. The cylindrical lower clamp was placed on an adjustable *x-y* table to allow precise vertical orientation of the humerus with respect to the tendon clamp. Gap formation was assessed using the MicroScribe M (Revware, Inc), which allows accurate measurement (accuracy of up to ±0.0508 mm) of the position of the tendon pins and the bone markers during cyclic loading.^34^ The MicroScribe M was calibrated prior to each experiment using an internal coordinate system fixed to the setup.

### Cyclic Loading

The tensile tests consisted of an initial nondestructive cyclic loading phase followed by a destructive pull-to-failure test.^8^ The loading parameters for both the cyclic and the pull-to-failure tests were selected according to previously published protocols.^21,32,41^ A 10-N preload was applied to the RCR constructs for 60 seconds before starting the 100 sinusoidal force-controlled loading cycles between 10 N and 30 N at a rate of 0.25 Hz. Positional measurements of the 4 markers were taken at time zero and at the end of cyclic loading, each with a preload of 10 N to remove potential slack. Gap formation was defined as the mean increase in distance (mm) between the anterior and posterior medial and lateral markers created during cyclic loading. This parameter was assessed by the change in position of the markers on the tendon relative to the fixed pins on the humerus. Peak-to-peak displacement was defined as the mean of the local minimum-maximum displacement during the 98th, 99th, and 100th cycles.^27^ Cyclic stiffness was calculated by averaging the stiffness of each individual cycle, determined using the least squares method. The first cycle of each loading series was not considered in these calculations.

### Tensile Testing to Failure

After cyclic loading, the specimens were linearly loaded to failure at a rate of 1 mm/s, starting from a preload of 10 N. From the load-deformation curve obtained, linear stiffness was defined as the slope of the linear portion between 20 N and 80 N, calculated using the least squares method. The failure force was defined as the maximal force that the specimens could withstand prior to a substantial drop.^21,31^ All calculations were performed in MATLAB R2020b (MathWorks). Additionally, the failure mode was recorded for each repair construct.

### Statistical Analysis

For statistical analysis, GraphPad Prism 9.5.1 (GraphPad Software) was used. An a priori power analysis was performed assuming similar performance to historical test data, which showed a mean of 24 ± 13 N and 122 ± 32 N for the control and interlocking patch–augmented RCR groups, respectively. Corresponding to an effect size of *d* = 4.0 for a *t* test and *F* = 1.5 for a 1-way analysis of variance (ANOVA) of 3 groups. The minimum sample size required was calculated to achieve a power of 80% with a 5% significance level. For a 2-tailed *t* test, a minimum sample size of N = 6 (n = 3 per group) would be required, and for a 1-way fixed-effects ANOVA for 3 groups, a minimum sample size of N = 9 (n = 3 per group) would be required. Each group was compared with every other group to identify significant differences among them. Assumptions of parametric data were evaluated to determine their validity. The normality of data distribution within groups was assessed using the Shapiro-Wilk test, where a significant test statistic indicated a deviation from normality. Homogeneity of variance was tested using the Levene test, with a significant result indicating that variances among groups were significantly different. If assumptions for parametric testing were met, 1-way ANOVA was conducted, followed by post hoc testing if the *F*-statistic was significant. For nonparametric data, the Kruskal-Wallis test was utilized.^43^

## Results

**Figure 3.**
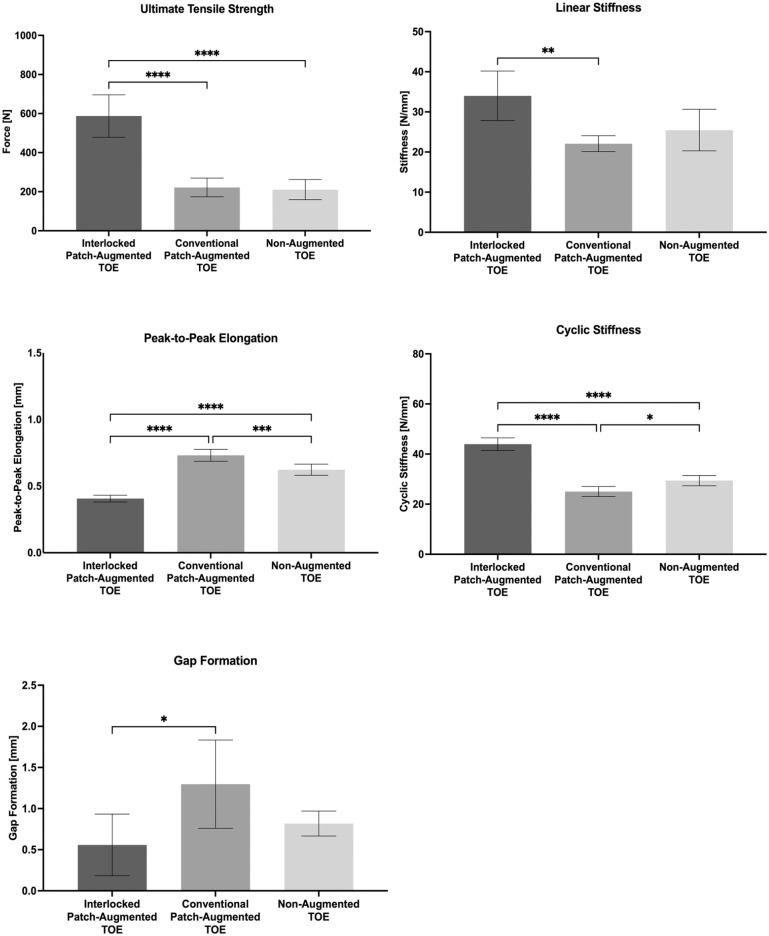
Results of cyclic loading and tensile testing. Plots show mean ± SD. *P < .05; **P < .01; ***P < .001; ****P < .0001.

The specimens were 6 to 8 months old, and none of the specimens had to be discarded. When compared with a nonaugmented RCR and a conventional patch–augmented RCR, a repair with the interlocking, nonwoven PET patch resulted in superior biomechanical properties including decreased gap formation (0.8 ± 0.2 mm and 1.3 ± 0.5 mm vs 0.6 ± 0.4 mm; *P* = .55 and *P* = .02), increased cyclic stiffness (29 ± 2 N/mm and 25 ± 2 N/mm vs 44 ± 3 N/mm; *P* < .0001 for both comparisons) and peak-to-peak elongation (0.6 ± 0.0 mm and 0.7 ± 0.0 mm vs 0.4 ± 0.0 mm; *P* < .0001 for both comparisons), as well as greater ultimate tensile strength during pull to failure (211 ± 52 N and 222 ± 48 N vs 587 ± 109 N; *P* < .0001 for both comparisons) and linear stiffness (25 ± 5 N/mm and 22 ± 2 N/mm vs 34 ± 6 N/mm; *P* = .28 and *P* = .007) (Table 1 and Figure 3). There was no significant difference between gap formation and linear stiffness of the nonaugmented RCR and the interlocking, nonwoven PET patch.

**Table 1:** Results of Cyclic Loading and Tensile Testing^*a*^

	Interlocked Patch–Augmented TOE (experimental)	Conventional Patch– Augmented TOE (control 2)		Nonaugmented TOE (control 1)	
	Mean ± SD	Mean ± SD	*P* [95% CI of difference]	Mean ± SD	*P* [95% CI of difference]
Ultimate tensile strength, N	587.3 ± 108.6	221.7 ± 48.1	**<.0001** [253.5 to 477.7]	210.5 ± 51.5	**<.0001** [264.7 to 488.9]
Linear stiffness, N/mm	34.0 ± 6.2	22.1 ± 2.0	.**007**	25.45 ± 5.2	.28
Cyclic stiffness, N/mm	43.9 ± 2.5	25.0 ± 2.0	**<.0001** [15.6 to 22.2]	29.4 ± 2.0	**<.0001** [11.3 to 17.9]
Gap formation, mm	0.6 ± 0.4	1.3 ± 0.5	.**02** [–1.34 to −0.13]	0.8 ± 0.2	.55 [–0.89 to 0.37]
Cyclic elongation, mm	0.8 ± 0.1	1.0 ± 0.2	.20 [–0.53 to 0.09]	1.0 ± 0.3	.48 [–0.45 to 0.17]
Peak-peak elongation, mm	0.4 ± 0.0	0.7 ± 0.0	**<.0001** [–0.38 to −0.27]	0.6 ± 0.0	**<.0001** [–0.27 to −0.16]

a Bold values indicate statistical significance; TOE, transosseous equivalent.

In relative terms, augmentation of the TOE with the directly interlocking, nonwoven PET patch compared with a nonaugmented TOE resulted in a 50% increase in cyclic stiffness, a 34% decrease in peak-to-peak elongation, and a 179% increase in ultimate tensile strength. Differences in gap formation and linear stiffness were not significantly different. Augmentation of the TOE with the directly interlocking, nonwoven PET patch compared with the conventional patch–augmented TOE resulted in a 57% decrease in gap formation, a 44% decrease in peak-to-peak elongation, a 76% increase in cyclic stiffness, a 54% increase in linear stiffness, and a 165% increase in ultimate tensile strength.

### Failure Mechanism

**Figure 4.**
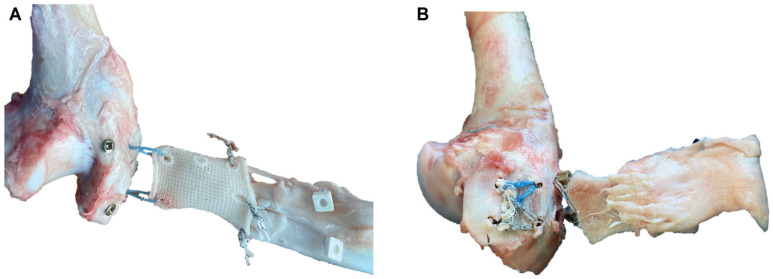
Photos were taken after pull-to-failure testing. (A) Bursal-sided view onto the tendon-patch interface of a conventional patch–augmented TOE. The white, rectangular markers were initially placed just medial to the conventional patch. The No. 2 Fiberwire sutures, which connected the patch to the tendon, tore through the tendon in line with the tendon fibers during pull-to-failure testing and there was little to no contact between the tendon and the patch anymore after pull-to-failure testing. Therefore, failure occurred at the “bone-tendon interface” as well as the patch-tendon interface. Note the distance between the white, rectangular markers and the patch. (B) Articular-sided view onto the tendon-patch interface of an interlocked patch–augmented TOE. Failure during pull-to-failure testing occurred at the bone-tendon interface, but the tendon remained in good contact with the interlocked patch. The quick selection and cutout features of Microsoft PowerPoint were used to enhance visualization. TOE, transosseous equivalent.

For all specimens, the failure site was at the tendon-bone and therefore the suture-tendon interface. For the interlocking PET patch specifically, the articular-sided portion of the tendon tore away from the footprint while the patch remained in contact and interlocked with the tendon. Additionally, partial detachment at the tendon-patch interface was observed in 2 cases, and in 1 case, there was pullout of a lateral patch anchor. In contrast, for the conventional patch group, the sutures connecting the tendon to the bone as well as the sutures connecting the patch to the tendon cut through the tendon simultaneously and there was little to no contact between the tendon and the patch at the time of failure (Figure 4).

## Discussion

The most important finding of the study was that augmentation of a conventional TOE with direct interlocking of a nonwoven PET patch yielded biomechanically superior results compared with both conventionally augmented and nonaugmented TOE RCRs. The direct interlocking of patch fibers into the underlying tendon enhanced the repair’s biomechanical properties: not only did this technique increase the time-zero force to failure to 265% compared with a conventional patch–augmented TOE and to 280% compared with a nonaugmented TOE, but it also improved linear stiffness to 154% and 135%, respectively. Higher construct stiffness suggests that micromotion and gap formation were minimized, an aspect that is crucial for tendon-bone healing and for reducing early tendon retraction, thereby offering potential to improve retear rates in future clinical applications.

In clinical practice, shoulder surgeons primarily use patches to augment RCRs when tendon quality is suboptimal, such as in degenerative tears or revision RCRs. In these situations, establishing solid contact between the tendon and the bone, and therefore strengthening the patch-tendon interface, within the first few weeks postoperatively is crucial to increase the likelihood of tendon healing. Early tendon retraction, within the first 6 weeks after surgery, correlates with formation of a recurrent tendon defect and worse clinical outcomes.^28^ Retear rates after RCR remain high, especially in chronic tears with degenerated tendons.^7,13,16^ The failure mode of RCRs usually involves sutures cutting through the tendon tissue, making the suture-tendon interface the most vulnerable part of the RCR, not only in primary repairs but also for patch-augmented repairs.^15,21,33^ Consequently, numerous groups have biomechanically evaluated various approaches for RCR augmentation, yet no consensus has emerged on a preferred technique. Although patch-augmented RCR generally results in lower retear rates compared with nonaugmented RCR, these rates still vary significantly. For synthetic patches, retear rates range from 9% to 70% over follow-up periods of 1 to 10 years.^3,11,39^ Similarly, allo- and autografts show retear rates between 8% and 15% after short-term follow-up of 2 years.^5,20,30^ Commercially available patches are typically sutured to the tendon, or in cases with short tendons, they are sutured to the myotendinous junction. We assume that this weak suture purchase is the main reason why they fail to increase linear stiffness of the repair construct. Improving linear stiffness, the resistance of a material to deformation under a single, constant load, is particularly important in the immediate postoperative period to ensure the tendon remains stable and properly positioned during healing.

Multiple patch materials and techniques have been explored, often achieving significant increases in ultimate load to failure. However, no patch consistently improved time-zero linear stiffness or cyclic stiffness, both of which are critical characteristics for successful tendon repair. Higher stiffness helps to maintain footprint contact by minimizing micromotion and preventing gap formation.^6,33^ Derwin et al^15^ evaluated the biomechanical and functional effect of an RCR augmentation using a poly-L-lactide (PLLA) scaffold in a partial RCT canine model. In Derwin et al’s study, 8 adult male mongrel dogs underwent bilateral shoulder surgery. One shoulder underwent tendon release and repair only, and the other was subjected to release and repair followed by augmentation with the repair device. Their study showed a significant increase in ultimate load to failure (796 ± 34 N vs 397 ± 18 N) but found no significant difference in stiffness at time zero (195 ± 2 N/mm) compared with nonaugmented controls. Similarly, Omae et al^33^ investigated the biomechanical performance of a repaired rotator cuff using an acellular human dermal matrix in human cadavers. They reported a significant improvement in ultimate load to failure (560 ± 96 N vs 346 ± 61 N) but a significant decrease in linear stiffness (65 ± 16 N/mm vs 77 ± 17 N/mm) for the augmented group. Furthermore, Jung et al^21^ examined the biomechanics of a conventional TOE repair compared with 3 augmented TOE repairs with different placements of a dermal collagen patch. They observed a significant increase in ultimate load to failure for the patch-augmented groups (226 N vs 140 N) but no significant differences in linear stiffness among the 4 groups. Comparing absolute values between studies is challenging due to differences in study designs (eg, creation of partial- vs full-thickness tears, animal models, underlying repair techniques). However, our interlocked patch not only significantly improved ultimate load to failure but also enhanced both cyclic and linear stiffness of the repair construct. In contrast, and consistent with the literature, conventional patch augmentation led to no significant difference in linear stiffness, but a significant decrease in cyclic stiffness compared with nonaugmented TOE RCR. We hypothesize that the extra sutures and the corresponding incisions in the tendon structure with conventional patch augmentation may contribute to a slight reduction in stiffness.

This surgical technique involved a felt patch attached to the tendon across the entire patch-tendon intersection, while other techniques involve a patch sutured only to specific locations.^9,17,18,25,26^ This robust attachment enhances load sharing between the tendon and the patch, resulting in enhanced suture retention properties^29^ and a greater overall strength of the repair construct. A previous study^29^ has demonstrated the excellent biocompatibility of the interlocking approach in vivo, showing successful adherence of native tissue and repair-site remodeling. Additionally, numerous studies have confirmed the biocompatibility of polymer patches,^12,22,35,40^ suggesting a favorable and potentially faster healing response, with desired ingrowth of tissue into the felt microstructure.

### Limitations

This study had several limitations. First, the evaluation was limited to the time-zero biomechanical properties of the repair construct. Therefore, no conclusions could be drawn regarding the mid- and long-term outcomes of the surgical technique or the biological reactions induced by it. Additional in vivo animal trials are necessary to analyze the biomechanical properties of the repair constructs at different stages of healing, evaluating the temporal effects of the patch augmentation. For instance, Derwin et al,^15^ who did not observe a significant increase in stiffness of repairs augmented with a PLLA scaffold at time zero, found a significant increase in stiffness at 12 weeks for the augmented repairs compared with the nonaugmented controls. Second, the results cannot be directly translated to humans because ovine animal models were used instead of human cadaveric shoulders. Although the sheep is a well-established animal model for rotator cuff studies, the immature ovine tendons do not accurately represent a chronic or degenerative tendon model. Furthermore, the force transmission applied along the native pull direction of ovine ISPs does not fully replicate the complex force transmission in physiological supraspinatus tendons. Third, gap formation was manually measured using the MicroScribe M with 2 medial tendon markers and 2 lateral bone pins. Although the MicroScribe M has a measurement accuracy of up to ±0.0508 mm, the variability in gap formation measurements was substantial, and results may have been biased by the manual placement of the pins and the compromised rigidity of the sutured tendon markers.

## Conclusion

While limiting but not eliminating tendon retraction, augmentation of a conventional TOE with direct interlocking of a nonwoven PET patch provided biomechanically superior results compared with conventionally augmented and nonaugmented TOE RCRs. The interlocked patch not only significantly improved time-zero force to failure but, compared with a conventional commercial patch design, also increased linear stiffness.

## Footnotes

‖ References 2, 14, 19, 21, 23, 24, 32, 41, 42.
